# Ethnicity and trial recruitment

**DOI:** 10.1186/cc14582

**Published:** 2015-03-16

**Authors:** HL Cronshaw, SW Scott, S Bowrey, JP Thompson

**Affiliations:** 1 Leicester Royal Infirmary, Leicester, UK

## Introduction

Surrogate consent and time-sensitive recruitment in critical care research is challenging, yet low enrolment numbers or omitting ethnic groups skew

## Results

and conclusions. Patients from different ethnic groups may respond differently to therapeutics [1]. There are few data about the effect of ethnicity on recruitment into ICU trials. Our ICU recruits to national trials and serves an increasingly non-White British population (24% of population). We undertook this study to determine whether ethnicity affects ICU consent rates.

## Methods

We performed a retrospective review of screening logs from three national UK trials (PROMISE, BALTI-P, GAiNS) and one local trial (Nociceptin in Sepsis). We analysed consent rates of eligible patients by ethnicity, age, sex, interventional or observational trial, and ethnicity of the researcher seeking consent. We performed chi-squared analysis, and entered significant values into a logistic regression model using SPSS v22.

## Results

We identified 332 eligible patients across all trials, of whom 37 (11%) were not White British (nWB). Analysis demonstrated consent/assent refusal being significantly associated with: nWB (14, 38%, *P *< 0.001), interventional trial (21, 25%, *P *= 0.003) and different researcher-patient ethnicities (*P *< 0.001). Logistic regression analysis confirmed these as independent factors (nWB OR = 4.5, 95% CI = 2.1 to 9.8, *P *< 0.001; interventional trial OR = 2.7, 95% CI = 1.4 to 5.2, *P *= 0.003; data points missing for researcher-patient ethnicity so variable excluded).

## Conclusion

This initial study suggests that ethnicity may affect assent/ consent to ICU research, with patients from different ethnicities being four times less likely to be recruited. Whilst data are incomplete for researcher-patient ethnicity, our data suggest that this may be an important factor and may influence future consent processes. We believe that the role of ethnicity warrants further investigation, not only in clinical trials but also in areas such as organ donation.
